# Brain function in classic galactosemia, a galactosemia network (GalNet) members review

**DOI:** 10.3389/fgene.2024.1355962

**Published:** 2024-02-15

**Authors:** Bianca Panis, E. Naomi Vos, Ivo Barić, Annet M. Bosch, Martijn C. G. J. Brouwers, Alberto Burlina, David Cassiman, David J. Coman, María L. Couce, Anibh M. Das, Didem Demirbas, Aurélie Empain, Matthias Gautschi, Olga Grafakou, Stephanie Grunewald, Sandra D. K. Kingma, Ina Knerr, Elisa Leão-Teles, Dorothea Möslinger, Elaine Murphy, Katrin Õunap, Adriana Pané, Sabrina Paci, Rossella Parini, Isabel A. Rivera, Sabine Scholl-Bürgi, Ida V. D. Schwartz, Triantafyllia Sdogou, Loai A. Shakerdi, Anastasia Skouma, Karolina M. Stepien, Eileen P. Treacy, Susan Waisbren, Gerard T. Berry, M. Estela Rubio-Gozalbo

**Affiliations:** ^1^ Department of Pediatrics, MosaKids Children’s Hospital, Maastricht University Medical Centre, Maastricht, Netherlands; ^2^ European Reference Network for Hereditary Metabolic Disorders (MetabERN) Member, Padova, Italy; ^3^ United for Metabolic Diseases (UMD), Amsterdam, Netherlands; ^4^ Department of Clinical Genetics, Maastricht University Medical Centre, Maastricht, Netherlands; ^5^ GROW School for Oncology and Reproduction, Faculty of Health, Medicine and Life Sciences, Maastricht University, Maastricht, Netherlands; ^6^ Department of Pediatrics, University Hospital Center Zagreb, Croatia, and School of Medicine, University of Zagreb, Zagreb, Croatia; ^7^ Department of Pediatrics, Division of Metabolic Diseases, Emma Children’s Hospital, Amsterdam University Medical Center, Amsterdam Gastroenterology Endocrinology Metabolism, Inborn Errors of Metabolism, Amsterdam, Netherlands; ^8^ Department of Internal Medicine, Division of Endocrinology and Metabolic Disease, Maastricht University Medical Centre, Cardiovascular Research Institute Maastricht (CARIM), Maastricht University, Maastricht, Netherlands; ^9^ Division of Inherited Metabolic Diseases, Reference Centre Expanded Newborn Screening, University Hospital Padova, Padova, Italy; ^10^ Laboratory of Hepatology, Department of Chronic Diseases, Metabolism and Ageing, Faculty of Medicine, KU Leuven, Leuven, Belgium; ^11^ Queensland Children’s Hospital, Children’s Health Queensland, Brisbane, QLD, Australia; ^12^ Department of Pediatrics, Diagnosis and Treatment Unit of Congenital Metabolic Diseases, University Clinical Hospital of Santiago de Compostela, IDIS-Health Research Institute of Santiago de Compostela, CIBERER, RICORS Instituto Salud Carlos III, Santiago de Compostela, Spain; ^13^ Department of Paediatrics, Pediatric Metabolic Medicine, Hannover Medical School, Hannover, Germany; ^14^ Division of Genetics and Genomics, Boston Children’s Hospital, Harvard Medical School, Manton Center for Orphan Disease Research, Boston, MA, United States; ^15^ Department of Paediatrics, Metabolic and Nutrition Unit, Division of Endocrinology, Diabetes and Metabolism, University Hospital for Children Queen Fabiola, Bruxelles, Belgium; ^16^ Department of Paediatrics, Institute of Clinical Chemistry, Inselspital, Bern University Hospital, Swiss Reference Centre for Inborn Errors of Metabolism, Site Bern, Division of Pediatric Endocrinology, Diabetes and Metabolism, University of Bern, Bern, Switzerland; ^17^ IEM Clinic, Arch Makarios III Hospital, Nicosia, Cyprus; ^18^ Metabolic Unit Great Ormond Street Hospital and Institute for Child Health, University College London, London, United Kingdom; ^19^ Centre for Metabolic Diseases, University Hospital Antwerp, University of Antwerp, Antwerp, Belgium; ^20^ National Centre for Inherited Metabolic Disorders, Children’s Health Ireland at Temple Street, University College Dublin, Dublin, Ireland; ^21^ Reference Centre of Inherited Metabolic Diseases, Centro Hospitalar Universitário São João, Porto, Portugal; ^22^ Department of Paediatrics and Adolescent Medicine, Medical University of Vienna, Vienna, Austria; ^23^ Charles Dent Metabolic Unit, National Hospital for Neurology and Neurosurgery (NHNN), London, United Kingdom; ^24^ Genetics and Personalized Medicine Clinic, Faculty of Medicine, Tartu University Hospital, Institute of Clinical Medicine, University of Tartu, Tartu, Estonia; ^25^ Endocrinology and Nutrition Department, Hospital Clínic de Barcelona, Centro de Investigación Biomédica en Red de la Fisiopatología de la Obesidad y Nutrición (CIBEROBN), Instituto de Salud Carlos III (ISCIII), Madrid, Spain; ^26^ Inborn Errors of Metabolism, Clinical Department of Pediatrics, San Paolo Hospital - ASST Santi Paolo e Carlo, University of Milan, Milan, Italy; ^27^ Rare Diseases Unit, Department of Internal Medicine, San Gerardo Hospital IRCCS, Monza, Italy; ^28^ iMed.ULisboa–Instituto de Investigação do Medicamento, Faculdade de Farmácia, Universidade de Lisboa, Lisboa, Portugal; ^29^ Department of Child and Adolescent Health, Division of Pediatrics I-Inherited Metabolic Disorders, Medical University Innsbruck, Innsbruck, Austria; ^30^ Medical Genetics Service, Hospital de Clinicas de Porto Alegre, Porto Alegre, Brazil; ^31^ Newborn Screening Department, Institute of Child Health, Athens, Greece; ^32^ Adult Metabolics/Genetics, National Centre for Inherited Metabolic Disorders, The Mater Misericordiae University Hospital, Dublin, Ireland; ^33^ Salford Royal Organisation, Northern Care Alliance NHS Foundation Trust, Salford, United Kingdom; ^34^ School of Medicine, Trinity College Dublin, National Rare Diseases Office, Mater Misericordiae University Hospital, Dublin, Ireland

**Keywords:** classic galactosemia, brain, galactose, cognitive problems, neurodevelopment, movement disorders, neuropsychiatry

## Abstract

Classic galactosemia (CG, OMIM #230400, ORPHA: 79,239) is a hereditary disorder of galactose metabolism that, despite treatment with galactose restriction, affects brain function in 85% of the patients. Problems with cognitive function, neuropsychological/social emotional difficulties, neurological symptoms, and abnormalities in neuroimaging and electrophysiological assessments are frequently reported in this group of patients, with an enormous individual variability. In this review, we describe the role of impaired galactose metabolism on brain dysfunction based on state of the art knowledge. Several proposed disease mechanisms are discussed, as well as the time of damage and potential treatment options. Furthermore, we combine data from longitudinal, cross-sectional and retrospective studies with the observations of specialist teams treating this disease to depict the brain disease course over time. Based on current data and insights, the majority of patients do not exhibit cognitive decline. A subset of patients, often with early onset cerebral and cerebellar volume loss, can nevertheless experience neurological worsening. While a large number of patients with CG suffer from anxiety and depression, the increased complaints about memory loss, anxiety and depression at an older age are likely multifactorial in origin.

## 1 Galactose metabolism

Galactose is a natural aldohexose that exists as free galactose and as a component of complex carbohydrates, glycoproteins and glycolipids. Together with glucose, galactose forms lactose, a disaccharide abundantly present in dairy products. Among other functions, it serves as a key source of energy in infants and is important for galactosylation of complex molecules such as galactocerebroside in myelin. On average, 88% of galactose is retained in the liver (Coelho et al., 2015a; Conte et al., 2021).

**FIGURE 1:**
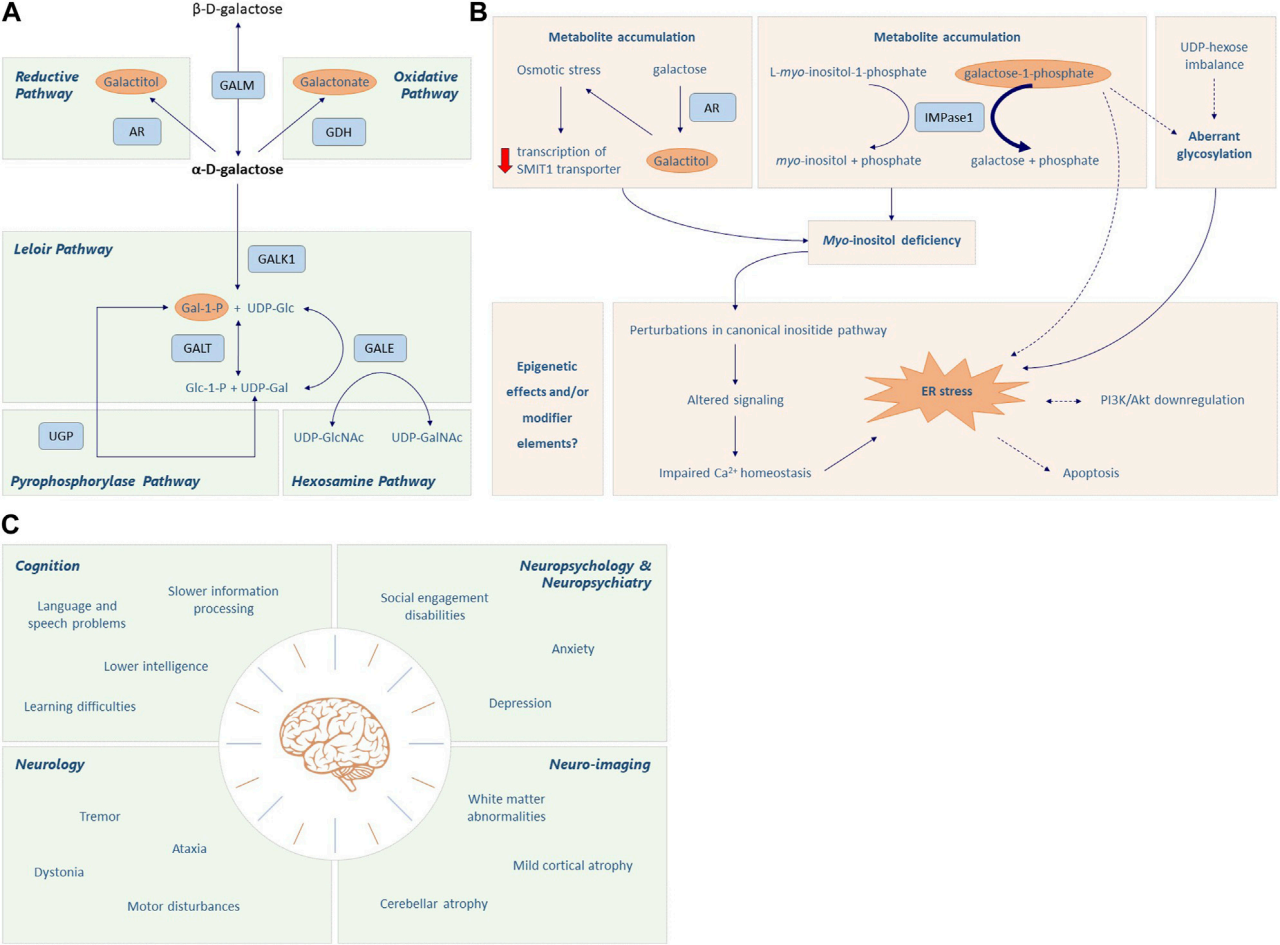
Galactose metabolism and CG pathophysiology. **(A)** The first step of the Leloir pathway involves the conversion of β-D-galactose to its stereoisomer α-D-galactose by galactose mutarotase (GALM). Then, α-D-galactose is phosphorylated to α-D-galactose-1-phosphate (Gal-1-P) by galactokinase (GALK1). Galactose-1-phosphate uridylyltransferase (GALT) catalyzes the 2-step reaction through which Gal-1-P and UDP-glucose are converted to α-D-glucose-1-phosphate and UDP-galactose. Finally, UDP-galactose 4′-epimerase (GALE) mediates the interconversion of UDP-galactose (UDP-gal) and UDP-glucose (UDP-glc). This enzyme is crucial to maintain the steady state UDP-galactose/UDP-glucose ratio in different cells, playing an important role in glycoconjugate formation. Accumulation of α-D-galactose due to GALT deficiency leads to the formation of galactitol and galactonate, mediated by aldose reductase (AR) and galactose dehydrogenase (GALDH), respectively. Additionally, Gal-1-P can be converted into UDP-Gal via the action of UDP-glucose pyrophosphorylase (UGP); however its affinity is much lower when compared to the main substrate, Glc-1-P. Except for GALK1, the enzymes in this pathway can work in both directions, depending on the substrate levels and energy demand of the cell. Please note that there are only two enzymes in humans that are capable of converting Gal-1-P to UDP-galactose, the GALT enzyme and the UGP enzyme. Additionally, while UGP is bidirectional in nature, the reaction usually goes in the direction of Glc-1-P to UDP-Glc because PPi is rapidly hydrolyzed. **(B)** The accumulation of toxic metabolites, aberrant glycosylation, *myo*-inositol deficiency, endoplasmic reticulum (ER) stress and oxidative stress, and signaling pathway alterations all seem implicated in the pathophysiological cascade elicited in CG. Increased levels of Gal-1-P can inhibit inositol monophosphatase (IMPase1), which converts L-*myo*-inositol-1-phosphate to free *myo*-inositol, thereby limiting the intracellular *myo*-inositol concentration. Accumulation of galactitol generates osmotic stress which may result in decreased transcription of the *myo*-inositol cotransporter SMIT1, further aggravating the intracellular *myo*-inositol deficiency. The *myo*-inositol deficiency and subsequent alterations in inositide signaling can impair calcium homeostasis and cause ER stress, which is associated with apoptosis and downregulation of PI3K/Akt signaling. Gal-1-P and aberrant glycosylation may also contribute to ER stress. Lastly, the role of epigenetics and modifier genes also needs to be considered in CG pathology. Dotted lines represent associations that are still under debate. **(C)** Classic Galactosemia (CG) patients can suffer from brain pathology in multiple domains, i.e., cognition, neurology, neuropsychology, neuropsychiatry and neuro-imaging.

The Leloir pathway is the main pathway of galactose metabolism and consists of four steps, consecutively mediated by galactose mutarotase (GALM EC 5.1.3.3), galactokinase (GALK1 EC 2.7.1.6), galactose-1-phosphate uridylyltransferase (GALT, EC 2.7.7.2012) and UDP-galactose 4′-epimerase (GALE, EC 5.1.3.7) (Figure 1A). Galactose entering the Leloir pathway either becomes a precursor for glycosylation (as UDP-galactose) or is used in glycolysis and glycogen synthesis pathways (as UDP-glucose) (Conte et al., 2021).

In Classic Galactosemia (CG), severe deficiency of GALT (<1% residual activity) fuels several alternative galactose disposal routes. Firstly, aldose reductase (AR, EC 1.1.1.21) converts α-D-galactose into galactitol in a NADPH-dependent reaction. Secondly, galactose is oxidized to galactonate by galactose dehydrogenase (GALDH, EC 1.1.1.48), producing NADH. Galactonate is excreted from the body or converted to D-xylulose 5-phosphate to enter the pentose phosphate pathway (Coelho et al., 2015a; Conte et al., 2021). Lastly, although a poor substrate (low affinity), Gal-1-P can be converted to UDP-galactose by UDP-glucose pyrophosphorylase 2 (UGP2, EC 2.7.7.9) (Coelho et al., 2015a).

## 2 Clinical spectrum

The first description of an infant with galactosemia dates from 1908 (von Reuss, 1908). In the following years, more patients were described with hypergalactosemia and neonatal illness, including hepatocellular damage, renal tubular disease, *Escherichia coli* sepsis, encephalopathy and cataract (Göppert, 1917; Mason and Turner, 1935). A well-recognized phenomenon is brain edema, also called ‘pseudotumor cerebri’, leading to increased intracranial pressure and bulging of the fontanel (Wells et al., 1965; Quan-Ma et al., 1966; Huttenlocher et al., 1970; Belman et al., 1986; Berry et al., 2001). A galactose-restricted diet resolves the acute neonatal symptoms but is insufficient to prevent long-term complications, which have the same prevalency in patients with and without neonatal illness.

Brain impairments occur in 85% of CG patients despite diet (Rubio-Gozalbo et al., 2019) (Figure 1C; Supplementary Table S1). Cognitive problems frequently experienced are global developmental delay and language delay (Komrower and Lee, 1970; Fishler et al., 1980; Waisbren et al., 1983; Waggoner et al., 1990; Schweitzer et al., 1993; Kaufman et al., 1995a; Hansen et al., 1996; Robertson et al., 2000; Antshel et al., 2004; Bosch et al., 2004; Potter et al., 2008; Hughes et al., 2009; Hoffmann et al., 2011; Potter, 2011; Timmers et al., 2011; Timmers et al., 2012; Waisbren et al., 2012; Potter et al., 2013; Demirbas et al., 2019; Kuiper et al., 2019; Welsink-Karssies et al., 2020a; Welsink-Karssies et al., 2020b; Hermans et al., 2023), with a below average mean total intelligence quotient (IQ) of 87 (Coss et al., 2013; Welling et al., 2017a). The language and speech impairments cannot solely be explained by lower cognitive abilities (Waisbren et al., 1983; Waggoner et al., 1990; Schweitzer et al., 1993; Kaufman et al., 1995a; Robertson et al., 2000; Antshel et al., 2004; Potter et al., 2008; Hughes et al., 2009; Potter, 2011; Timmers et al., 2011; Timmers et al., 2012; Waisbren et al., 2012; Potter et al., 2013; Kuiper et al., 2019). Expressive language is mainly affected, with receptive language and comprehension being relatively preserved (Potter et al., 2008; Timmers et al., 2011). Among the speech disorders are verbal dyspraxia (23.5%) and dysarthria (19.9%) (Nelson et al., 1991; Potter et al., 2008; Potter, 2011; Kuiper et al., 2019). Patients require more time to prepare and finish the utterances and make more errors (Timmers et al., 2012), and also recruit additional and more extensive brain regions than control participants (Timmers et al., 2015a).

Approximately half of the CG patients suffer from neurological complications, the most prevalent being tremor (31.0%), which may affect daily life in some cases (Rubio-Gozalbo et al., 2019). Other complications include general motor abnormalities, ataxia, dystonia and epilepsy (Jan and Wilson, 1973; Lo et al., 1984; Bohles et al., 1986; Friedman et al., 1989; Waggoner et al., 1990; Koch et al., 1992; Schweitzer et al., 1993; Robertson et al., 2000; Arn, 2003; Antshel et al., 2004; Martins et al., 2004; Ridel et al., 2005; Potter et al., 2008; Hughes et al., 2009; Shah and Kuchhai, 2009; Waisbren et al., 2012; Rubio-Agusti et al., 2013; Demirbas et al., 2019; Kuiper et al., 2019; Rubio-Gozalbo et al., 2019; Welling et al., 2019; Özgün et al., 2019; Welsink-Karssies et al., 2020a; MacWilliams et al., 2021). Epilepsy is not frequently reported (Friedman et al., 1989; Aydin-Ozemir et al., 2014) and may be the result of brain damage occurring in the neonatal period, or the consequence of unrelated genetic predisposition. Psychiatric and behavioral problems such as depression and anxiety disorder are reported in 44.4% of the patients (Rubio-Gozalbo et al., 2019). Most have a shy and reserved personality (Antshel et al., 2004) and achieve fewer social developmental milestones when compared to healthy controls, which is postulated to be intrinsic to the disease rather than a result of the burden of a chronic disease or lifelong dietary restrictions (Bosch et al., 2009; Gubbels et al., 2011).

Numerous central nervous system (CNS) grey and white matter abnormalities have been reported in CG (Crome, 1962; Haberland et al., 1971; Lo et al., 1984; Choulot et al., 1991; Koch et al., 1992; Nelson et al., 1992; Kaufman et al., 1995b; Hughes et al., 2009; Timmers et al., 2015b; Timmers et al., 2016; Özgün et al., 2019; Ahtam et al., 2020; Welsink-Karssies et al., 2020a; Welsink-Karssies et al., 2020c). Magnetic resonance imaging (MRI) in a cohort of 67 patients showed cerebral and cerebellar atrophy in 22 and 8 patients, respectively, as well as white matter abnormalities in 11 patients (Nelson et al., 1992). In a study that assessed the integrity of myelinated networks, abnormal somatosensory evoked potentials were present in 17 (28%) of 60 CG patients who had electrophysiological testing of the median nerve, and in 26 (77%) of 34 CG patients who had the posterior tibial nerve tested (Kaufman et al., 1995b). Neurite orientation dispersion and density imaging (NODDI) revealed a lower neurite density index (NDI) in bilateral anterior areas and increased orientation dispersion index (ODI) mainly in the left hemisphere (Timmers et al., 2015b). More recent studies showed lower white matter volume and impaired microstructure in the whole brain, especially in the corticospinal tract (Welsink-Karssies et al., 2020c), as well as the left cerebellum, bilateral putamen and left superior temporal sulcus (Ahtam et al., 2020). Additional disturbances in grey matter density have also been described (Nelson et al., 1992; Dubroff et al., 2008; Timmers et al., 2016; Ahtam et al., 2020).

Several studies have attempted to correlate the grey and white matter abnormalities with clinical outcome. The severity of symptoms at the age of diagnosis was associated with abnormal somatosensory evoked potentials (Kaufman et al., 1995b). Additionally, neurocognitive outcome was linked to patients’ resting-state brain connectivity patterns (van Erven et al., 2017), and grey matter density disturbances associated with later initiation of dietary intervention (Timmers et al., 2016). Furthermore, patients with a tremor and/or dystonia had smaller white matter volume, more impaired white matter microstructure and less myelin compared to patients without movement disorders. Patients with IQ < 85 had grey and white matter abnormalities, as well as lower cerebral and cerebellar volume (Welsink-Karssies et al., 2020c). Lastly, language difficulties were correlated with abnormal diffusivity values of the bilateral dorsal and ventral language networks (Ahtam et al., 2020).

Although the aforementioned abnormalities could help explain the neurocognitive profile, the possibility of a coexistent disorder should always be considered, especially in case of unexpected symptoms (Papachristoforou et al., 2014; Neville et al., 2016; Boca and Whone, 2017; Rossi-Espagnet et al., 2021).

## 3 Proposed disease mechanisms

Toxic metabolites, aberrant glycosylation, *myo*-inositol deficiency, endoplasmic reticulum (ER) stress and oxidative stress, signaling pathway alterations, and structural impairment of GALT, all seem implicated in the pathophysiological cascade elicited in CG (Haskovic et al., 2020) (Figure 1B). Additionally, the role of epigenetics and modifier genes needs to be considered. Different mechanisms could be acting synergistically, depending on the tissue type and developmental stage.

### 3.1 Metabolite toxicity

Despite diet, the levels of galactose metabolites are persistently increased due to endogenous production of galactose, which is mainly derived from lysosomal hydrolysis of glycolipids, glycoproteins and proteoglycans (Berry et al., 1995a; Berry et al., 1997). The rate of galactose production is higher in infants and children and decreases until adulthood (Berry et al., 2004; Schadewaldt et al., 2004).

Gal-1-P is deemed one of the key pathogenic agents of CG (Gitzelmann, 1995; Leslie, 2003; Lai et al., 2009). Toxicity has been ascribed to the inhibition of enzymes like UGP, phosphoglucomutase, glycogen phosphorylase and inositol monophosphatase, but convincing evidence is still lacking (Gitzelmann, 1995; Lai et al., 2009). Notably, GALK1 deficiency, which causes accumulation of the galactose metabolites except Gal-1-P, does not give rise to the brain and ovarian complications seen in CG (Tang et al., 2010).

Galactitol excretion in urine can be elevated up to 300 times in patients on diet (Krabbi et al., 2011). Studies have reported galactitol elevations in the brains of neonatal and pediatric CG patients (Quan-Ma et al., 1966; Berry et al., 2001; Otaduy et al., 2006; Rossi-Espagnet et al., 2021). Galactitol is poorly diffusible and highly osmotic, and can lead to cell swelling and brain edema. *In vivo* elevation of brain galactitol was associated with diffuse white matter abnormalities in a newborn with CG and encephalopathy (Berry et al., 2001). Furthermore, increased T2 signal in white matter and areas of restricted diffusion involving the cortex and deep grey matter nuclei, consistent with cytotoxic edema, was observed in 3 patients during neonatal illness and confirmed galactitol accumulation (Rossi-Espagnet et al., 2021).

Little attention has been paid to the possible role of galactonate in CG pathophysiology. Although the metabolite is excreted in urine or used in the pentose phosphate pathway, its toxicity cannot be ruled out completely and requires further study (Berry et al., 1998).

### 3.2 Aberrant glycosylation

Aberrant glycosylation has been hypothesized to be a major mechanism of disease (Maratha et al., 2017). UDP-hexoses serve as key sugar donors for glycosylation, and deficiency of UDP-galactose and disturbance of the UDP-glucose/UDP-galactose ratio have been described in CG (Charlwood et al., 1998; Lai et al., 2003; Coss et al., 2014; Maratha et al., 2017). Furthermore, Gal-1-P may compete as substrate for other nucleotide sugar reactions.

Systemic glycan assembly defects have been documented in neonatal illness which largely resolve with galactose restriction (Charlwood et al., 1998; Quintana et al., 2009). However, there is evidence of continuing glycan processing abnormalities (Coss et al., 2012; Coss et al., 2014; Maratha et al., 2017). Of interest, in a CG sibling study, marked differences in outcomes of the second born siblings were noted, with early onset cerebellar and cerebral atrophy in 2 sibling pairs (Hughes et al., 2009), and significant differences in N-glycosylation in later life (Coman et al., 2010).

While differences in glycosylation can be identified in CG individuals at older age, with differing tolerances to moderate galactose intake liberalization (Coss et al., 2012; Knerr et al., 2015), the significance of these findings is unknown. Polymorphic glycan modifier genes (*MGAT3*, *FUT8* and *ALG9*) can influence glycan chain bisecting and fucosylation, and subsequent cell signaling and adhesion (Wahl et al., 2018).

Myelin may be especially vulnerable to disturbed glycosylation, as it is rich in galactocerebrosides (Barnes-Vélez et al., 2023). Low levels in autopsy brain tissue of an untreated patient raised the question of aberrant glycosylation of galactocerebrosides (Haberland et al., 1971; Koch et al., 1992; Lebea and Pretorius, 2005). Glycosylation also plays an important role in the neuromuscular junction (NMJ) (Dani and Broadie, 2012), and GALT was identified as a potent regulator of NMJ structure in *Drosophila melanogaster* (Jumbo-Lucioni et al., 2014).

### 3.3 *Myo*-inositol deficiency

*Myo*-inositol serves a dual role in human physiology. It is a precursor of membrane phospholipids that are important for calcium- and protein kinase C signaling, and serves as a buffer of osmotic balance (Berry et al., 1995b; Berry, 2011). Brain content of *myo*-inositol peaks prenatally and continues to decline until a postnatal baseline is reached, which is maintained up to a second decline at middle age (Kreis et al., 2002; Buccafusca et al., 2008). Reduction in intracellular *myo*-inositol has been associated with impaired integrated stress response signaling and ER stress (Wells and Remy, 1965; Slepak et al., 2007; Hagen-Lillevik et al., 2022). The first reports of *myo*-inositol deficiency in the brain of CG children date back to 1965 (Wells et al., 1965) and 1966 (Quan-Ma et al., 1966). High levels of Gal-1-P may sequester *myo*-inositol as inositol monophosphate by inhibition of inositol monophosphatase (Slepak et al., 2007). In addition, galactitol accumulation may lead to poor *myo*-inositol transport into the cell, further decreasing *myo*-inositol availability (Berry, 2011).

### 3.4 Endoplasmic reticulum stress, oxidative stress and signaling pathway alterations

ER stress (Slepak et al., 2007; De-Souza et al., 2014) and oxidative stress (Slepak et al., 2007; Jumbo-Lucioni et al., 2012; Tang et al., 2014) are two other pathological mechanisms. In fibroblasts derived from CG patients (Slepak et al., 2007) and *GalT* gene-trapped mice (Balakrishnan et al., 2016; Balakrishnan et al., 2017), evidence was found for activation of the unfolded protein response and ER stress. Interestingly, salubrinal (an eIF2α phosphatase inhibitor) administration in these mice reversed the downregulation of PI3K/Akt signaling pathway and significantly slowed down the loss of Purkinje cells in the cerebellum (Balakrishnan et al., 2017). Additionally, administration of purple sweet potato color (PSPC) and *myo*-inositol, two compounds hypothesized to rescue aberrant signaling pathways in CG partly due to their antioxidant properties, ameliorated dysregulation of cellular pathways in this model (Hagen-Lillevik et al., 2022).

### 3.5 Structural impairments of GALT

The fundamental biochemical cause of the disease is a severe decrease in enzymatic activity. Some of the pathogenic variants result in a less stable protein that is unable to reach a correct folding, and so has an increased propensity to aggregation and proteolysis (McCorvie et al., 2013; Coelho et al., 2014).

### 3.6 Potential epigenetic effects and genetic modifiers

The role of epigenetics and modifier genes needs to be studied more extensively. Genetic modifiers are genetic variants that can influence the phenotypic outcome of a disease-causing variant in another gene, and have repeatedly been postulated to explain the phenotypic variability seen in CG (see also subsection aberrant glycosylation). It is well recognized that genetic modifiers can affect glycosylation pathways in Congenital Disorders of Glycosylation, rendering what was considered to be single gene abnormalities as ‘multifactorial’ (Quelhas et al., 2023). Several genetic modifiers have already been discovered for other rare Mendelian disorders (Rahit and Tarailo-Graovac, 2020).

## 4 Time of damage

Intra-uterine toxicity of galactose metabolites has been postulated an important pathogenic factor (Holton, 1995; Segal, 1995). Gal-1-P was elevated in the liver of galactosemic fetuses at 20 weeks gestation, as well as in the cord blood of galactosemic infants born to mothers who abstained from galactose consumption during pregnancy (Gitzelmann, 1995; Holton, 1995).

GALT activity measured in several animal models throughout development (Shin-Buehring et al., 1977; Rogers et al., 1989a; Rogers et al., 1989b; Rogers et al., 1992; Daude et al., 1996) was particularly higher in the early postnatal period relative to adulthood, which has been attributed to the high galactose ingestion and physiological needs during the suckling period (Rogers et al., 1989a). *GALT* mRNA and protein are already weakly expressed during late embryonic and postnatal development of the brain and peripheral nerve of the rat, with a peak of expression concomitant with myelogenesis (Daude et al., 1996). GALT activity in the late prenatal stage in various organs of a sheep model (Coelho et al., 2017) showed that galactosemia acute target organs–liver, small intestine and kidney–had the highest late prenatal activity, whereas the chronic target organs–brain and ovary–did not exhibit a noticeable pre- or postnatal different activity, in line with the notion that some organs/cells have a greater susceptibility to impaired galactose metabolism.

Supporting an early life injury, disruptions of fiber tracts and brain nuclei formed during embryogenesis and early fetal brain development were reported in ten adult patients (Ahtam et al., 2020). Moreover, a recent study that used retinal neuro-axonal imaging as a surrogate of brain pathology to assess neuronal integrity and monitor neurodegenerative disease progression pointed towards early brain damage (Lotz-Havla et al., 2023).

Some movement disorders, e.g., tremor, are more frequently seen at an older age (Kuiper et al., 2019). It is not clear whether disease-related mechanisms continue to damage structures (striatum/cerebellum) or whether this is the result of a prenatal/perinatal hit with a dying back phenomenon. Vasogenic edema might play a role in the delayed myelination later in life (Rossi-Espagnet et al., 2021). However, disturbances in myelination are also found in children without neonatal illness, suggesting the implication of other disease mechanisms.

## 5 Follow-up/treatment current and future perspectives

Dietary galactose restriction is currently the cornerstone for treatment but does not prevent long-term complications. In 2016, the members of the Galactosemia Network (GalNet) developed an evidence-based and internationally applicable guideline for diagnosis, treatment and follow-up of CG patients (Welling et al., 2017b). The guideline recommends a galactose-restricted diet that eliminates sources of galactose from dairy products but permits galactose from non-milk sources. The natural history study showed that patients with a liberalized diet did not have a worse outcome neurologically (Rubio-Gozalbo et al., 2019). Moderate liberalization of galactose intake improved IgG glycosylation in a small number of patients (Coss et al., 2014; Knerr et al., 2015). The guideline also offers guidance for testing various neurocognitive and psychosocial domains to facilitate tailored interventions as part of the treatment plan.

In search of new therapeutic approaches, extensive research has been performed to limit accumulation of toxic metabolites or increase levels of deficient metabolites (Simard-Duquesne et al., 1985; Ng et al., 1989; Mizisin and Powell, 1993; Tang et al., 2012; Ji et al., 2017; Hu et al., 2019; Mackinnon et al., 2021). GALK1 inhibitors were shown to prevent accumulation of Gal-1-P in cellular models, but remain to be studied *in vivo* (Tang et al., 2012; Hu et al., 2019; Mackinnon et al., 2021). Uridine supplementation to increase levels of UDP-Glc and UDP-Gal was not able to rescue the biochemical and clinical phenotype (Ng et al., 1989). Safety and effectiveness of the AR inhibitor AT007 is currently being investigated (NCT04902781; NCT05418829).

Furthermore, to improve GALT activity, chaperone therapy and nucleic acid therapy have been studied. Supplementation of the amino acid arginine as a chaperone had a mutation-specific effect with rescue of human *GALT* in an *E. coli* model (Coelho et al., 2015b), but failed to exhibit positive effects in four c.563A>G; p.Gln188Arg homozygous patients (Haskovic et al., 2018). Whether other pathogenic variants are amenable has not been studied. *hGALT* mRNA therapy and *GALT* gene therapy restored GALT activity in cellular and animal models of CG (Balakrishnan et al., 2020; Brophy et al., 2022; Daenzer et al., 2022; Delnoy et al., 2022), but many unknowns remain to be answered before these therapies can be applied. Other treatment options could be compounds that target the integrated stress response such as PSPC and *myo-*inositol (Balakrishnan et al., 2016), which improved brain tissue structures in *GalT* gene-trapped mice (Hagen-Lillevik et al., 2022). An advantage is their favorable safety profile in humans, which could hasten their application in clinical practice.

## 6 Brain function through adulthood

In CG, brain function is affected in 85% of the patients, with significant individual variability in severity and symptoms (Rubio-Gozalbo et al., 2019). Since Komrower (Komrower and Lee, 1970) in 1970 reported on physical and mental development in the first cohort of 60 dietary treated CG patients, numerous studies have been performed to shed more light on brain effects in CG (summarized in Supplementary Table S1). While these have expanded our knowledge on the neurocognitive, neuropsychological, neuropsychiatric, social emotional and neurological difficulties associated with the disease, as well as the abnormalities seen in neuroimaging and electrophysiological assessments, information about the disease course in adulthood is still scarce. The majority of studies are cross-sectional, or retrospective using cross-sectional data from different age groups. The few longitudinal studies that have been performed are usually based on small sample sizes, with relatively young patients and limited follow-up time. Although the results from these studies should be treated cautiously, they provide us with the first valuable insights into CG brain function over time.

Nelson et al. (Nelson et al., 1992) performed MRI imaging in 63 CG patients (1 month–42 years of age). Of the 24 patients who underwent follow-up MRI after 1–4 years, abnormal peripheral white matter and ventricle enlargement remained unchanged. One patient showed progression of cerebellar atrophy. Schadewaldt et al. (Schadewaldt et al., 2010) reported TIQ, PIQ and VIQ scores in 23 patients, with the first tests performed at a mean age of 11±5 years and the second tests at a mean age of 26±5 years. The mean TIQ and PIQ did not change significantly over time, whereas the mean VIQ score showed a variable but significant decline at follow-up. However, no consistent changes were found, as a number of participants showed significant increases and other patients decreases of these scores with age. In line with these results, neurocognitive function did not deteriorate in a cohort of 35 patients aged 1 week - 16 years, but the follow-up time was only 2–5 years (Manis et al., 1997). A recent study in a robust dataset of CG patients (mean age of 18 years) concluded that speech/voice/language, cognitive, motor, and psychosocial outcomes are not progressive in most patients, but also here the time between testing was limited (Smith et al., 2023). A pilot study with 10 adult patients (mean age 33 years) and a mean time interval of 3 years and 9 months reported cognitive stability (Hermans et al., 2024).

In addition to the longitudinal studies, Lotz-Havla et al. (Lotz-Havla et al., 2023) studied retinal neuroaxonal function as marker for neurodegeneration in 11 CG patients and 60 controls, and did not find evidence for retinal neuroaxonal degeneration. Moreover, specialist teams within the GalNet that treat CG patients and take part in this review observe improvements in language performance (scores on verbal tests), and absence of cognitive decline. Patients, nevertheless, do complain about motor and social function, and at older ages complaints about tremor, memory issues, anxiety and depression are reported more often.

Although there might be progression concerning signs of early aging, memory issues and depression, “growing into deficits” can play a role. Adult life is often more stressful than childhood, so the features of anxiety and depression (financial worries, loneliness) may be more pronounced with time. There is also a subset of patients that experience neurological worsening, which are very often patients who already had significant neurological issues in childhood. Patients with early onset cerebral or cerebellar atrophy can also show progressive natural senescence effects. The pathophysiological mechanisms therefore seem multifactorial, with individual susceptibility as one of the most important determinants. Studies with sensitive tests for the different affected domains and follow-up of decades in larger cohorts need to be performed to adequately delineate the disease course through adulthood.

## 7 Conclusion

In this review, we describe the role of impaired galactose metabolism on brain dysfunction. Our conclusion is that, based on the current data and insights, the majority of patients do not exhibit cognitive decline. A subset of patients experiences neurological worsening, often those patients with early onset cerebral and cerebellar volume loss. At older ages complaints about memory issues, anxiety and depression are seen more often, but are likely multifactorial in origin.

## Abbreviations

AR, aldose reductase; CG, classic galactosemia; CNS, central nervous system; ER, endoplasmic reticulum; Gal-1-P, galactose-1-phospate; GALDH, galactose dehydrogenase; GALE, UDP-galactose 4’-epimerase; GALK1, galactokinase; GalNet, Galactosemia Network; GALT, galactose-1-phosphate uridylyltransferase; GALM, galactose mutarotase; IQ, intelligence quotient; MRI: magnetic resonance imaging; NDI, neurite density index; NMJ: neuromuscular junction; NODDI, neurite orientation dispersion and density imaging; ODI, orientation dispersion index; PIQ, performance IQ; PSPC, purple sweet potato color; TIQ, total IQ; UDP, Uridine diphosphate; UGP2, UDP-glucose pyrophosphorylase 2: VIQ, verbal component IQ.
