# Supplementary material for: Brain function in classic galactosemia, a galactosemia network (GalNet) members review
Source: Front Genet. 2024 Feb 15;15:1355962. doi: 10.3389/fgene.2024.1355962 (PMC10902464; doi:10.3389/fgene.2024.1355962)
Supplement: Supplementary file 1 [file Table1.docx]

**Table 1: Summary of studies on neurocognitive domains and neuropsychological/social emotional abnormalities, neuroimaging, electrophysiology and neurologic symptoms in CG (longitudinal, cross-sectional and retrospective studies).**

| **Longitudinal studies: neurocognitive domains and neuropsychological/social emotional abnormalities** | | | | |
| --- | --- | --- | --- | --- |
| **Reference** | **Number of patients included** | **Study design/**  **method** | **Time of follow up** | **Summary /conclusions** |
| Manis, F.R., et al., J Inherit Metab Dis, 1997. **20**(4): p. 549-55 | 35 patients  (18 m, 17 f), age range 1 week-16.2 years | Neuro-cognitive testing | 2-5 years | No support for any developmental change in cognitive functioning over time for this sample. None of the patients showed evidence of progressive declines in overall cognitive ability |
| Schadewaldt, P., et al., Pediatrics, 2010. **125**(2): p. e374-81 | 23 patients  11 f, 12 m. First IQ test at a mean age of 11 +/- 5 years and the second 13.6 to 15.5 years later at a mean age of 26 +/- 5 years | Neuro-  cognitive testing | Mean interval between testing 14.9 ± 0.4 years | Mean scores in the first and second tests:   - TIQ, 78 ± 14 (73 [58–106]) and 73 ± 15 (70 [55–109]), not statistically different - PIQ, 73 ± 17 (70 [45–103]) and 74 ± 17 (68 [47–111]), not statistically different - VIQ, 86 ± 11 (84 [69–113]) and 77 ± 13 (76 [57–105]), the mean VIQ score showed a variable but statistically significant decline (P < 0.001; mean: −9 ± 10 [range: −33 to 16 points]) - In the first and second tests, TIQ scores in the average range of intellectual performance (85–115) were observed in 7 and 5 patients, respectively, and TIQ scores were below average in 16 and 18 patients, respectively |
| Smith, N.H., et al., Mol Genet Metab, 2023. **140**(3): p. 107708 | 159 children and adults (90 f, 69 m, mean age 18.1 years (range 0.5-76.9 years) | Cross-sectional and longitudinal data of neuro-cognitive testing | At least 12 months | Cross sectional: speech outcome affected in 58/124 (46.8%), cognitive outcome affected in 51/121 (42.1%), motor outcome affected in 38/115 (33.0%), psychosocial outcome affected in 30/112 (26.8%)  No progression in speech, voice, language, cognitive, motor and psychosocial outcomes in most of the patients, in both cross-sectional and longitudinal analyses, neither in children nor in adults |
|  |  |  |  |  |
| Hermans, M.E. et al., JIMD Rep, 2024. **1**: 1-6 | 10 patients with IQ ≥ 70 or independent work or living situation (8 f, 2 m). Mean age 33 years, range 22-49 years | Longitudinal data of neuro- psychological testing | Mean time interval of 3 years and 9 months (range 1023-1575 days) | No significant decline or improvement in test scores on all neuropsychological measures (verbal and visual information processing speed executive function (i.e. cognitive flexibility inhibition), verbal fluency and visuospatial functioning, except a decline on the Trail Making Test-A (p = 0.048) |
| **Longitudinal studies: neuroimaging** | | | | |
| Nelson, M.D., et al*.,* Radiology, 1992. **184**(1): p. 255-61 | 67 patients (36 f, 31 m) galactosemia patients, median age 10 years, range 1 months to 42 years. 63/67 CG | Cross-sectional and longitudinal data of MRI | In 24 patients follow-up MRI 1-4 years after the initial examination | Patients < 1 year: 8/8 normal white matter signal intensity  Patients ≥ 1 year: 52/55 abnormalities in peripheral cerebral and cerebellar white matter signal  Patients ≥ 1 months-42 years: cerebellar atrophy 8/63, in which 6 mild, and 2 moderate  Patients ≥ 1 months-42 years: multiple small hyper intense lesions in the cerebral white matter on T2-weighted images 11/63 (cluster in corners of lateral ventricles)  Patients ≥ 1 months-42 years: ataxia in 10/63  Patients ≥ 1 months-42 years: ventricle enlargement (cerebral atrophy) in 22/63  Patients < 2 years old: 9/12 underwent follow-up imaging 1-2 years after initial imaging: all 9 abnormal peripheral myelination  Patients 2-25 years: 15 patients underwent follow-up imaging 1-4 years after initial imaging: abnormal peripheral white matter pattern more evident, unchanged on the follow-up imaging  Of the patients with ventricle enlargement (cerebral atrophy), 22/63: 4 underwent follow-up examinations 1-2 years after initial imaging: no change in enlargement  Of the 24 patients who underwent follow-up imaging: 1/24 progression of cerebellar atrophy |
| **Cross-sectional and retrospective studies: neurocognitive domains and neuropsychological/social emotional abnormalities** | | | | |
| Komrower, G.M. and D.H. Lee*.,* Arch Dis Child, 1970. **45**(241): p. 367-73 | 60 children (22 m, 38 f) | Physical examination and neuro-psychological testing | IQ range: 30-118 (mean of 80). The boys (2.2-13.9 years) averaged 89 and the girls (2.3-17.9 years) averaged 73. The IQ scoring for the children in the 'good dietary control' group was 84, range 51-118 (average) compared with 77, range 51-107 for the 'moderate to poor dietary control' group. They found that a rigid dietary control does not play a significant beneficial role in respect of intelligence and intellectual development   - Age group 0-5 years: mean IQ 90, range 61-118 (n=21) - Age group 5-10 years: mean IQ 79, range 30-112 (n=25) - Age group 10-15 years: mean IQ 70, range 45-107 (n=14)   High scores on scales like depression, lack of assertiveness, withdrawn and anxious towards adults in 30 patients | |
| Fishler, K., et al., Clin Pediatr (Phila), 1980. **19**(1): p. 38-44 | 60 patients, 31 f, 29 m from 47 families. Age range 5 months-29.5 years | Neuro-psychological testing, EEG and visual-perceptual tests | Age 0-5.5 years (n=13): Developmental Quotient/Intelligence Quotient: mean 102, standard deviation 12.8, range 70-125, 4/4 normal visual-perceptual status, 4/5 normal EEG, 1/5 abnormal EEG  Age 6-16 years (n=25): Developmental Quotient/Intelligence Quotient mean 91, standard deviation 17.5, range 50-117, 7/25 normal and 18/25 abnormal visual-perceptual status, 12/24 normal EEG, 12 abnormal EEG/24  Age 17-29 years (n=22): Developmental Quotient/Intelligence Quotient mean 94, standard deviation 18.2, range 72-119, 14/22 normal and 8/22 abnormal visual-perceptual status, 17/22 normal EEG, 5/22 abnormal EEG | |
| Waisbren, S.E., et al*.,* J Pediatr, 1983. **102**(1): p. 75-7 | 8 children, age range 3.6-11.6 years. 6 m, 2 f | Psychological and speech / language testing | Psychological tests Mean performance score 102 ± 11  Mean full-scale IQ 97 ± 13  Mean verbal score 92 ± 14  Speech and language Speech and language deficits in at least 1 category: 7/8  Receptive language deficit 4/8  Articulation deficit 5/8  Expressive language deficit 4/7  Short term memory deficit 5/6  Neurological symptoms: 1/8 seizures, 1/8 mild hemiparesis | |
| Waggoner, D.D., et al.,*.* J Inherit Metab Dis, 1990. **13**(6): p. 802-18 | 350 patients, mean age 9.5 years (range 2 weeks-37 years) 51% m, 49% f | Questionnaire | Developmental quotient < 1 years: mean 109 ± 11, range 89-136 (n=35)  Developmental quotient 1-2 years: mean 97 ± 17, range 45-123 (n=71)  Intellectual quotient 3-5 years: mean 92 ± 19, range 50-138 (n=85)  Intellectual quotient 6-9 years: mean 87 ± 19, range 39-142 (n=88)  Intellectual quotient 10-16 years: mean 80 ± 17, range 26-115 (n=67)  Intellectual quotient > 16 years: mean 81 ± 21, range 20-110 (n=29)  Speech problems in 136/243 (56%), with 92% delayed vocabulary, 90% articulation problems, 65% received speech therapy  Motor problems including gait problems, and coordination problems in 37/206 (18%): coordination problems in 26/206, gait problems in 14/206, balance problems in 7/206, fine motor tremor in 9/206 and severe ataxia in 2/206 | |
| Nelson, C.D., et al. 1991., Pediatrics, 1991. **88**(2): p.346-350 | 24 patients, 11 m, 13 f, age range 3.3-14 years | Neuro-psychological examination, speech evaluation | Verbal dyspraxia: 13/24 (54%)  Articulation disorders without verbal dyspraxia: 2/24 (8%)  Normal articulation: 9/24 (38%)  IQ scores of patients with dyspraxia: mean 79 ± 18 SD and IQ scores of patients without dyspraxia: 99 ± 13 (P<0.01)  Presence or severity of dyspraxia not related to age at which diet was begun, presence of neonatal symptoms, gender, age at time of speech evaluation | |
| Bosch, A.M., et al*.,* Pediatrics, 2004. **113**(4): p. e423-e428 | 63 patients, 24 m, 39 f. Age range 1-41 years | Questionnaire | Age 1-5 years: more abdominal complaints and communication problems  Age 8 to 15 years: more problems on domain of cognitive function  Mothers of patients aged 6 to 15 years reported a significantly lower quality of life on the domains of motor and cognitive function  Patients ≥16 years: significant lower scores on the domains of cognitive and social function | |
| Antshel, K.M., et al., Neuro-psychology, 2004. **18**(4): p. 658-64 | 25 patients, mean age 10.7 years, range 8-14 years, 15 m, 10 f | Abbreviated neuro-psychological testing | Full scale IQ mean 84.3 (SD 8.3), versus controls (99.0, SD 15.5)  Verbal comprehension index mean 82 (SD 8.0), versus controls (101.8, SD 13.9)  Perceptual organization index mean 92.7 (SD 9.9) versus controls (96.9, SD 15.8)  Patients exhibited less well-developed executive functions compared to controls  Parents viewed their children as exhibiting more internalizing symptoms (e.g., depression, anxiety), but children viewed their emotional status differently | |
| Bosch, A.M., et al*.,* J Inherit Metab Dis, 2009. **32**(6): p. 706 | 15 adults (3 m, 12 f), age range 18-35 years (mean 24.2 years, SD 3.9) | Questionnaire | In patients with CG:   - Delayed social and psychosexual development - Significantly less frequently married or living together - Significantly less frequently employed | |
| Doyle, C.M., et al.*,* J Inherit Metab Dis, 2010. **33**(5): p. 603-9 | 28 patients (20 f, 8 m), mean age 29.3 years (range 15-53) | Neuro-psychologic examination | CG patients experienced impaired memory, language processing, visual-motor skills, and increased anxiety  Verbal IQ mean 88.9, SD 16.8  Performance IQ mean 85.1, SD 12.9 | |
| Gubbels, C.S., et al., J Inherit Metab Dis, 2011. **34**(2): p. 415-9 | 18 males (age 18-35 years) | Questionnaire | Severe delays in the social and psychosexual domains but not on the autonomy domain | |
| Hoffmann, B., et al., J Inherit Metab Dis, 2011. **34**(2): p.421-427 | 32 patients (12 f, 20 m). Mean age 21.2 ± 7.2 years (range 9.9-37.4 years) | Speech performance and cognitive test and neurological examination | Mean IQ 76.2 ± 14.8  Speech test: no impairments in 5/32 (15.6%, 3 children and 2 adults), in 84% errors in speech test  Speech errors: more related to pseudo-words than real words, predominantly observed in words with three and four syllables. The performance in producing words was correlated to the IQ scores | |
| Potter, N.L. J., Inherit Metab Dis, 2011. **34**(2): p. 377-85 | 33 children, age range 4-16 years | Speech and language testing | Motor speech disorder in 9/33 (27%)  Childhood apraxia of speech  (CAS) in 8/33 (21%)  Ataxic dysarthria in 1/33 (3%) and mixed CAS-ataxic dysarthria in 1/33 (3%)  Maximum phonation time less in CG by an average of 32%  Decreased respiratory-phonatory support for speech in 58%  Disturbed vocal quality indicative of cerebellar dysfunction in 33%  Vocal tremors in 3/33 (9%)    IQ score < 85: language disorder in 17/33 (8%), both receptive and expressive  IQ score > 85: language disorder in 16/33 (56%) typically expressive language only | |
| Jumbo-Lucioni, P.P., et al., J Inherit Metab Dis, 2012. **35**: p.1037-49 | 234 patients from 5 continents and 11 countries | Questionnaire survey | Diagnosis < 14 days of age: speech problems 43/114, neurological problems in 30/166, cognitive problems in 132/160, social problems in 65/98  Diagnosis > 14 days of age: speech problems 8/31, neurological problems in 11/32, cognitive problems in 8/31, social problems in 2/10 | |
| Timmers I., et al., PLoS One, 2012. **7**(12): p. e52826. | 22 patients (15 f, 7 m), mean age 14.9 years (range 10.8-19.1 years | Language production measured with event-related potentials (ERPs) | Lexical and syntactic planning of language production: CG patients need more time to prepare and finish the utterances and make more errors | |
| Potter, N.L., et al., JIMD Rep, 2013. **11**: p. 31-41 | 32 children, age range 4-16 years, 21 m, 11 f, with neurologic speech disorders | Speech and coordination assessment. Case control study | IQ range 85-115: 49%  IQ range 70-84: 39%  IQ range <70: 12%  In CG with neurologic speech disorders:   - More articulation errors, weaker hand and tongue strength - Coordination disorders (balance and manual dexterity): ≤ 5^th^ percentile in 17/32 (53%), < 10^th^ percentile in 21/32 (66%) - Children with childhood apraxia of speech (n = 7) and ataxic dysarthria (n = 1), had poorer balance and manual dexterity, but not weaker hand or tongue strength, compared to the children with fewer speech errors | |
| Coss, K.P., et al., J Inherit Metab Dis, 2013. **36**(1): p. 21-7 | 130 patients, age range 0.5-45 years | Retrospective descriptive study of the epidemiology of cases detected by newborn screening since 1972 | IQ tested (n=85), median age 18.8 years, range 6.0-39 years:   - IQ < 70 in 26/85 (30.6%) - IQ 70-79 in 22/85 (25.9%) - IQ 80-89 in 21/85 (24.7%) - IQ 90-109 in 10/85 (11.8%) - IQ 110-119 in 6/85 (7.1%)   Speech or language impairments (verbal dyspraxia, speech delay or difficulties, no speech or severe speech delays or verbal comprehension problems) in patients ≥ 2.5 years: 49.6 %  Ataxia in patients ≥ 2.5 years 7/117 (6.0 %) | |
| Timmers, I., et al., Brain Res, 2015. **1616**: p. 166-76 | 12 patients, 3 m, 9 f, median age 17.4 years, range 14.6-21.1 years | Functional magnetic resonance imaging (fMRI) | Altered neural activity and connectivity during active language performance. Patients recruit additional and more extensive brain regions when compared to controls  Special education in 75%  Speech therapy in 92%  Motor therapy in 42% | |
| Welling, L., et al., Disabil Rehabil, 2019. **41**(22), 2663-8 | 44 patients, 18 m, 26 f, median age 15 years, range 2-49 years | Interview | 4/44 (age 11-19 years) motor function impairment with mild to severe tremor, in 2/4 wheelchair bound.  3/44 (adults) abnormal sensory function (vision, hearing, tactile sense)  18/21 adults were living independently or were be able to live independently  9/44 (5 children and 4 adults) impairment of speech and voice functions | |
| Welsink-Karssies, M.M., et al., Orphanet J Rare Dis, 2020. **15**(42) p.1-13 | 48 patients, median age 16 years, range 4-47 years. 28 f, 20 m | Neuro- psychological testing and questionnaire | Full scale IQ 45-103 (mean 77 ± 14)  Visual IQ mean 82 ± 15  Performance IQ mean 78 ± 15  Lower but highly variable scores on all cognitive domains, especially on tests requiring mental speed, executive and visuospatial functioning  Social functioning scores were normal | |
| Robertson, A., et al., Genet Med, 2000. **2**(2): p. 142-8 | 113 patients. Average age 13.6 years (range 3-41 years), 51 m, 62 f | Questionnaire | Developmental verbal dyspraxia in 43/113 (38.1%) | |
| Hermans, M.E., et al., Orphanet J Rare Dis, 2023. **18**(1); p. 1-16 | 61 patients, mean age 22.1 years, range 1-52 years, 32 f, 29 m | Online self- and proxy questionnaire | Children 8-18 years: statistically more reports of fatigue, lower function in upper extremities  Parents of CG patients, age 5-18 years reported lower quality of peer relationships of their children and more fatigue  Both children and parents reported lower cognitive functioning  Adults reported lower cognitive functioning, higher anxiety and more fatigue | |
| Hermans, M.E., et al., Acta Neuropsychiatr, 2024. Jan 5; 1-12 | 23 patients (aged 8-52 years) without intellectual disability (TIQ ≥ 70) and/or independent work- or living situation, 14 females, 9 males | Neuro-psycho-logical testing, facial emotion recognition, Theory of Mind and self- reported empathy | In 22 patients: mean IQ 81.8 (range 69-103, SD 8.9), 68% TIQ < 85.  Children showed lower visual information processing speed, letter fluency and visuospatial functioning  Adult performed significantly lower on visual and verbal information processing speed, inhibition, letter fluency and visuospatial functioning  CG patients without intellectual disability: satisfied with their social competence, especially social functioning  Both children and adults significantly lower recognition of the emotions disgust, fear, happiness and sadness  Deficits in social cognition present (i.e. emotion-recognition, Theory of Mind and (self-reported) empathy  Social cognition has the largest impact on social competence in this sample of CG patients  Large variability in scores and discrepancies between self-and proxy-report | |
| **Cross-sectional and retrospective studies: neuroimaging** | | | | |
| **Reference** | **Number of patients included** | **Study design/**  **method** | **Summary/conclusions** | |
| Dubroff, J.G., et al*.,* J Inherit Metab Dis, 2008. **31**(4): p. 533-9 | 5 patients, mean age 28 years, range 20-40 years | FDG-PET scan* | All patients had neurological symptoms: tremor in 5/5, cerebellar ataxia in 2/5, speech problems in 3/5, dystonia in 3/5  Decreased cerebral glucose metabolism in the superior temporal gyrus, medial occipital lobe, parietal lobe, cerebellum, calcarine cortex, superior frontal cortex, and superior parietal cortex. Increases in cingulate gyrus and temporal poles, bilaterally. Foci of decreased glucose metabolism in the caudate, cerebellum, precentral gyrus and cerebellar tonsils and increased glucose metabolism in the subcallosal gyrus and claustrum. Abnormalities in cortical metabolism | |
| Timmers, I., et al., J Inherit Metab Dis, 2015. **38**(2): p. 295-304 | 8 patients (range 15,9-21.2 years),  2 m, 6 f | MRI | Microstructure abnormalities (abnormal density and orientation dispersion of axons) in line with language and motor abnormalities, and general profile of higher order cognitive impairments. Neurite density index was lower in bilateral anterior areas, and orientation dispersion index was increased mainly in the left hemisphere | |
| Timmers, I., et al*.,* Brain Res, 2016. **1648**(Pt A): p. 339-344. | 8 patients, 2 m and 6 f, mean age 17.9 years, range 15-21 years | Voxel-based morphometry MRI | A decreased grey matter density (bilaterally) in putamen, part of the subcortical nuclei of the basal ganglia and in the bilateral [occipital cortex](https://www.sciencedirect.com/topics/neuroscience/occipital-lobe). An increased grey matter density in inferior frontal and medial prefrontal cortex of both hemispheres. Affected regions in line with neuro-cognitive profile, including motor (speech) abnormalities, language production impairments and memory and executive functioning deficits Furthermore high correlations of grey matter regions with visual working memory, age and age of initiation of diets | |
| Van Erven, B., et al., Scientific Reports, 2017. **7**: p.1-13 | 12 patients (3 m, 9 f), median age 17.4 years, range 14.6-21.1 years | Functional MRI | Patients showed alterations in networks of:  - Medial prefrontal cortex, parietal lobule and (pre)cuneus, involved in spatial orientation and attention  - In insula, superior frontal gyrus, important for sensory-motor integration for motor (speech) planning.  - In occipital regions, linked to visuospatial capacities and working memory  Altered functional connectivity to the superior frontal cortex, anterior insula, parietal lobule and the (pre)cuneus was found that correlates with neurocognitive test results | |
| Welsink-Karssies, M.M., et al., Brain Commun, 2020. **2**(1): p. 1-16 | Total group: 56 patients with GALT activity <15%, 26 m and 30 f. Median age 18 years (range 0-48 years). Of these patients, 47 with GALT enzyme activity < 3.3% (“classical patients group”), 23 m and 24 f. Mean age 21 years (range 4-48 years) | Cohort study. MRI and clinical investigation and measurement of Gal-1-P and IgG N-glycan | Classical patients group: IQ median 77 (range 45-103), in 27/40 (68%) < 85, in 17/29 (59%) movement disorders  For the total group: IQ median 78 (range 45-103), in 29/47 (62%) < 85, in 17/36 (27%) movement disorders  Total group  tremor in 13/36 (36%), tremor + dystonia in 4/36 (11%) , no significant differences in N-glycans or Gal-1-P in patients with IQ < 85 and IQ ≥ 85  Brain abnormalities in 18/21 patients (86%) more severe in patients with IQ < 85 and/or movement disorders: 9/21 (43%) focal white matter abnormalities in frontoparietal region, in none focal abnormalities in infratentorial white matter, basal ganglia, thalamus and cortex. In 12/21 (57%) cerebral and cerebellar atrophy, with parietal in 16/17 (94%), frontal 9/17 (53%) and vermis atrophy in 13/17 (76%)  Abnormal gross motor skills in 9/43 (21%), abnormal fine motor skills in 5/41 (12%)  Abnormal speech and language development in 25/43 (58%) with language delay in 11/25 (44%) and speech defect in 4/25 (16%) and in a combination of both in 7/25 (28%) | |
| Welsink-Karssies, M.M., et al., Mol Genet Metab, 2020. **131**(4): p. 370-9 | 21 patients, 9 m, 12 f, median age 22 years, range 8-47 years | Cohort study with MRI | With Diffusion Tensor Imaging (DTI) and quantitative T1 mapping: White matter of CG patients: lower in volume and impaired microstructure in whole brain and corticospinal tract. Less myelin, more pronounced in 9/16 patients with tremor and/or dystonia  In 15/21 patients with an IQ < 85: grey matter and white matter of cerebrum and cerebellum more affected in volume when compared to 6/21 patients with an IQ ≥ 85. | |
| Rossi-Espagnet, M.C., et al*.,* Am J Neuroradiol, 2021. **42**(3): p. 590-6 | 17 patients, 15 m and 2 f; age range, 13 days to 14 years | MRI and MR spectroscopy | Imaging in distinct phases in CG  1) acute neonatal presentation, with predominant white matter edema  2) subacute clinical onset with “double cap sign”  3) a chronic phase with heterogeneous imaging findings | |
| Lotz-Havla, A.S., et al., J Clin Med, 2023. **12**(5): p.1-13 | 11 patients age 17–48 years, mean age 27.3 ± 8.5 years, 5 m, 6 f | Retinal spectral–domain optical coherence tomography | No signs of diminished peri-papillary retinal nerve fiber layer thickness and combined ganglion cell and inner plexiform layer volume  Speech disorder 8/11 (73%)  Ataxia 3/11 (27%)  Seizures 1/11 (9%)  Intellectual disability 6/11 (55%) | |
| **Cross-sectional and retrospective studies: electrophysiology** | | | | |
| Kaufman, F.R., et al*.,* J Child Neurol, 1995. **10**(1): p. 32-6 | 60 patients (27 m, 33 f), age range 6 months to 44 years | Median and posterior tibial nerve evoked potential, MRI and clinical, psychologic tests | Abnormal median nerve evoked potentials in 17/60 (28%): central slowing in 14 and peripheral neuropathy in 3  Abnormal posterior tibial nerve evoked potentials In 26/34 (77%): central slowing in 12, peripheral neuropathy in 14  Evoked potentials correlated with severity of symptoms, age at testing, presence of focal white matter lesions on MRI, but not with neurophysiological testing  MRI: abnormal white matter signal in 54/56 of patients > 1 year of age (96%), mild cerebral atrophy in 9, abnormal ventricular size in 22 and focal white matter lesions in 11  Tremor, ataxia and dysmetria in 12/60 | |
| **Cross-sectional and retrospective studies: neurological symptoms** | | | | |
| Schweitzer, S., et al., Eur J Pediatr, 1993. **152**(1): p. 36-43 | Clinical evaluation: 83 patients (34 f, 49 m, age 9 months to 33 years, mean age 9.5 ± 7.1 years)  Retrospective evaluations: 31 patients (16 f, 15 m, age 9 months-27 years, mean age 10.2 ± 8.8 years) | 83 patients (78 CG) by clinical psychometric testing, 31 by medical history evaluations | Clinical examination: Ataxia 6/78 (age of onset 9-14 years), clumsiness 12/78 intention tremor 11/78, microcephaly in 10/78, 1/78 epilepsy. In the medical history evaluations: normal neurological examination in 7/11, 3/11 epilepsy  Speech disturbances in 43/66 (65%) with 24/66 dyslalia, 9/66 dyspraxia, 7/66 dysgrammatism, 1/66 stuttering, in the medical history evaluation group: 12/31 speech impairments  Disturbances of visual perception and/or arithmetic deficits in 29/66, calculation deficits in 29/66, and in the retrospective group 7/31 calculation deficits, 3/31 disturbances in visual perception  IQ < 85 (no correlation between age of initiation therapy and or therapy compliance):   - Age < 6 years: 4/34 (12%) - Age 7-12 years: 10/18 (56%) - Age >12 years: 20/24 (83%) | |
| Kaufman, F.R., et al*.*, Eur J Pediatr, 1995. **154**(7 Suppl 2): p. S2-5 | 45 children (age range 4-18 years) and adults (18-39 years), 22 f, 23 m | Examination and MRI | Abnormal neurologic examination with ataxia, tremor and dysmetria in 12/45 (26%). No correlation between neurologic outcome (ataxia, tremor and/or dysmetria), genotype/phenotype and Q188R mutation  MRI cerebrum: White matter signal intensity in 37/40 (93%), cerebral atrophy in 7/40 (18%), abnormal large ventricular size in 16/40 (40%), focal white matter lesions in 8/40 (20%). No correlation between MRI findings, neurologic symptoms, age at diagnosis, severity of illness, cognitive outcome.  Cognitive functioning:   - - IQ 90-115 in 7/40 (17.5%)   - IQ 80-89 in 8/40 (20%)   - IQ 70-79 in 11/40 (27.5%)   - IQ < 70 in 14/40 (35%) | |
| Hansen, T.W.R., et al., Acta Paediatr, 1996. **85**: p.1197-1201 | 8 patients, age range: 9 months-19 years, 3 f, 5 m. Time of diagnosis between 2 and 11 weeks | Neurological examination, neuro-psychological testing, EEG, CT cerebrum | Low developmental scores in 5/8  Affected speech development in 5/8, with verbal dyspraxia in 3/6  Abnormal CT scan in 3/8  EEG abnormal in 1/8 with generalized slowing and theta activity without seizure components  1/8 cerebellar symptoms with nystagmus, dysdiadochokinesia and ataxia and intention tremor  1/8 gross and fine motor clumsiness. | |
| Waisbren, S.E., et al., J Inherit Metab Dis, 2012. **35**(2): p. 279-86 | 33 adults,  17 m and 16 f. (mean age 32.6 ± 11.7 years; range 18-59) | Examination, psychological testing, speech and language tests, subset EEG (n=6)  Subset health quality of life interview (n=13) | Full scale intelligence quotient (FSIQ): mean of 88 ± 20, range 55-122   - Scores ≤ 85 in 13/33 (39%) - Scores ≤ 70 in 8/33 (24%)   Executive functioning deficits in 5/33 (15%), low adaptive behavior assessment scores in 7/33 (21%), 15/33 lived independently (46%)  Diminished tongue strength, in 24/33 (73%). Decreased phonation duration in 21/33 (64%) and articulation proficiency reduced in 4/33 (12%)  Dysarthria in 8/33 (24%). Apraxia of speech in 3/33 (9%) Receptive vocabulary was reduced in 14/33 (42%) al).  Tremor in 15/33 (46%), intention tremor 8/33 (24%) postural tremor 5/33 (15%) and both postural and intention tremor in 2/33 (6%) ataxia in 5/33 (15%), dystonia in 2/33 (6%)  Life time reported depression in 13/33 (39%) and anxiety in 22 (67%)  Event-related potentials (ERPs): brain’s reaction to a language tasks: errors in 20.8% vs. 6.4% in controls, voice onset latency: 5.5 seconds vs. 1.5 seconds in controls, both different at p< 0.001. | |
| Rubio-Agusti, I., et al*.,* Mov Disord, 2013. **28**(6): p. 804-10 | 47 patients, median age 26 years, range 20-38 years, 29 f, 18 m. Median age at diagnosis was 14 days, range 7-17 days. | Direct examination and interview. Medical history examination | Motor dysfunction in 31/47 (66%)  Tremor in 23/47 (50%) with arm tremor in 23/31, postural tremor in 19/31, kinetic tremor in 12/31, and rest tremor in 2/31, head tremor in 4/31, leg tremor in 1/31  Dystonia in 23/47 (50%) with generalized dystonia in 4/31, segmental dystonia in 10/31, focal dystonia in 5/31, multifocal dystonia in 4/31  Cerebellar signs in 6/47 (13%)  Pyramidal signs in 4/47 (9%)  Epilepsy 3/47 (7%)  Cognitive impairment in 14/47 (30%)  Psychiatric symptoms in 11/47 (23%), anxiety in 5, depression in 4, obsessive-compulsive disorder in 3, autistic spectrum disorder in 2, social phobia in 1, paranoid delusions in 1  Speech problems in 16/47 (34%)  Brain MRI (6 patients with motor dysfunction, segmental dystonia): 3 normal  3 abnormal: 2 patients(dystonia, cerebellar and pyramidal signs): mild pontocerebellar atrophy, diffuse subcortical and pontocerebellar atrophy and bilateral symmetrical confluent signal change in periventricular white matter, 1 patient (generalized dystonia): signal changes and volume loss in cortex of the right posterior insula and supramarginal gyrus | |
| Rubio-Gozalbo, M.E., et al., Orphanet J Rare Dis, 2019. **14**(1): p. 1-11 | 509 patients (48% m, 52% f); age ranged from 0 to 65 years (median 18 years) | Observational study from web-based patient registry | Developmental delay 167/320 (52.2%)   - Motor developmental delay 18/167 (10.8%) - Cognitive delay 66/167 (39.5%)   Neurological complications 168/323 (52%)   - Tremor 104/336 (31%) - Ataxia 40/329 (12.2%) - Seizures 26/320 (8.1%) - Dystonia 24/318 (7.5%)   Language and speech disorder 192/289 (66.4%)   - Speech defects 129/315 (41%) - Impairment in vocabulary 117/288 (40.6%) - Impairment in grammar 98/253 (38.7%) - Verbal dyspraxia 67/285 (23.5%) - Dysarthria 49/246 (19.9%)   Mental (psychiatric) & behavioral problems 128/288 (44.4%)   - Anxiety disorder 67/300 (22.3%) - Depression 38/303 (12.5%) - ADHD 21/286 (7.3%) - Autism spectrum disorder 17/281 (6.0%) | |
| Demirbas, D., et al., Mol Genet Metab, 2019. **126**(4): p.368-376 | 135 patients, 67m, 68 f, classified as 0% (n=81), 0.1-1.0% (n=9), 1-13% (n=7) and 13-38% (n=38) GALT enzyme activity | Retrospective data, GALT enzyme measurement | In the 0% GALT activity: IQ mean 88.5 ± 17.9 (n=49), developmental delay 26/55 (47%), language delay 27/39 (69%), speech defect 39/67 (58%), dysarthria 11/46 (24%), ataxia 5/63 (8%), dystonia 5/46 (11%), tremor 18/70 (26%)  In the 0.1-1% GALT activity: IQ 52,76,80 (n=3), developmental delay 2/8 (25%), language delay 3/7 (43%), speech defect 6/9 (67%), dysarthria 0/7 (0%), ataxia 0/8 (0%), dystonia 0/9 (0%), tremor 2/9 (22%)  In the 1-13% GALT activity: IQ 129, 87 (n=2), developmental delay 2/6 (33%), language delay 3/5 (60%), speech defect 1/7 (14%), dysarthria 0/5 (0%), ataxia 0/6 (0%), dystonia 0/7 (0%), tremor 0/7 (0%)  In the 13-38% GALT activity: IQ 114 (n=1), developmental delay 3/33 (9%), language delay 5/24 (21%), speech defect 3/20 (15%), dysarthria 0/18 (0%), ataxia 0/29 (0%), dystonia 0/29 (0%), tremor 1/30 (3%) | |
| Kuiper, A., et al., J Inherit Metab Dis, 2019. **42**(3): p. 451-458 | 37 patients, 15 m, 22 f  Age < 18 years: 18/37 (48.6%) mean age 10.7 years  Age ≥ 18 y 19/37 (52.4%) mean age 32.9 years | Videotaped neurological and psychological examination and questionnaires | Motor disorder 18/37 (48.6%)   - Age <18y: 38.9% - Age >18y: 57.9%   Dystonia   - Age <18y: 3/7 - Age >18y: 9/11   Tremor   - Age <18y: 1/7 - Age >18y: 5/11   Myoclonus   - Age <18y: 4/7 - Age >18y: 0/11   Ataxia   - Age <18y: 1/7 - Age >18y: 3/11   Lifetime diagnosis of psychiatric disease: 8 (21.6%)  Autism spectrum disorder 4/8  Attention deficit hyperactivity disorder 3/8  Depression 2/8  Generalized anxiety 1/8  Psychotic episode 1/8  Behavioral problems in 47.2 %, internalizing problems most frequent in 38.9% (proxy-report) or 26.9% (self-report): most contributing subdomain: withdrawn/depressed in 33.3% and anxious/depressed in 16.7% (self-report) | |
| Özgün, N., et al., Int J Dev Neurosci, 2019. **78:** p.92-7 | 46 patients, 26 m, 20 m, mean age at neurological testing: 48.5 ± 28.5 months and of developmental testing 34.4 ± 18.2 months | Retrospective study | Convulsions in 11 (23.9%) patients (age not mentioned)  Neurological examination abnormal in 11/46 (24%) with ataxia in 6, tremor in 2, chorea and dystonia in 1, hypotonia in 1, horizontal nystagmus in 1  Delay in at least one developmental domain in 45.7%  Language developmental deficit in 41.3%  Abnormal MRI in 22/46 (47.8%), mean age of MRI 37.7 ± 17.7 months   - Signal intensity in periventricular white matter in 36.3% - Ventricular enlargement in 31.8% - Thinning of corpus callosum in 22.7% - Delayed myelination in 9% - Cerebellar atrophy in 9% - Hypo myelination in 9% - Cerebral atrophy in 4.5% - Mega cisterna magna in 13.6 % | |
| Ahtam, B., et al., J Inherit Metab Dis, 2020. **43**(6): p. 1205-18 | 10 patients, 5 m, 5 f (mean age 27.2 ± 5.5 years | Neurological and neuro-psychological evaluation, EEG (n=10) MRI (n=9) | Tremor 6/9 (67%) and dysarthria in 5/9 (56%), ataxia in 4/9 (40%) and abnormal gait in 6/9 (67%)  EEG abnormalities in 6/10: slowing 6/9  MRI: reduced volume of left cerebellum white matter, bilateral putamen, left superior temporal sulcus, impaired language networks  In CG impaired memory, language processing, visual-motor skills, and increased anxiety.  Mean full scale IQ was 80.4 ±  17.3. Verbal IQ mean 88.4 | |
| MacWilliams, J., et al., J Inherit Metab Dis, 2021. **44**(4): p. 871-8 | 57 patients, mean age 20.8 ± 12.4 years, 25 f, 32 m | Digital Archimedes spiral drawings on a touch screen | Digital Archimedes spirals: tremor (4-8 Hz) in 51% of patients versus 10% of controls  Self-reported history of speech difficulties in 27/52 | |
| * FDG-PET scan: [(18)F]fluorodeoxyglucose (FDG) positron emission tomography (PET) scan. F: female, m: male  The categorization of the articles is arbitrary, some studies belong to more categories.  Disclaimer: Although we have tried to include all studies published, it is possible that some studies are missing from the table. | | | | |
